# Influence of the programmed cell death of lymphocytes on the immunity of patients with atopic bronchial asthma

**DOI:** 10.1186/1710-1492-10-14

**Published:** 2014-03-19

**Authors:** Cyrille Alode Vodounon, Christophe Boni Chabi, Ylia Valerevna Skibo, Vincent Ezin, Nicolas Aikou, Simeon Oloni Kotchoni, Simon Ayeleroun Akpona, Lamine Baba-Moussa, Zinaida Ivanovna Abramova

**Affiliations:** 1 Laboratory Acid Nucleic, Institute of Fundamental Medicine and Biology, Kazan Federal University (KFU-Russian), Kremlyovskaya str. 18, Kazan 480008, Republic of Tatarstan, Russian Fédération; 2 Laboratoire de Biologie et de Typage Moléculaire en Microbiologie, Département de biochimie et biologie cellulaire, Faculté des sciences et Techniques (FAST), Université d’Abomey-Calavi (UAC-Benin), 05PB1604 Cotonou, Benin; 3 Department of Biology and Center for Computational & Integrative Biology, Rutgers University, Camden, NJ 08102, USA; 4 Laboratoire de Biochimie et Biologie Moléculaire, Faculté de Médecine, Université de Parakou, BP: 123 Parakou, Parakou, Benin

**Keywords:** Programmed cell death, Immunity, Atopic Bronchial Asthma

## Abstract

**Background:**

Fairly recent data highlight the role of programmed cell death and autoimmunity, as potentially important factors in the pathogenesis of chronic obstructive airway diseases. The purpose of our research was to determine the influence of apoptotic factors on the immunity of patients with atopic bronchial asthma according to the degree of severity.

**Method:**

The study was performed on the peripheral blood of patients with atopic bronchial asthma with different severity. The Immunological aspects were determined with ELISA, the fluorimetric method and the method of precipitation with polyethylene glycol. And the quantification of the parameters of the programmed cell death was performed by the method of flow cytometry and electron microscopy method.

**Results:**

The data obtained from morphological and biochemical parameters show the deregulation of Programmed Death of lymphocytes of patients with atopic bronchial asthma but individual for each group of patients. This dysfunction might induce the secretion of autoantibodies against DNA. This could explain the accumulation of circulating immune complex with average size considered as the most pathogenic in patients with bronchial asthma especially in the patients of serious severity. It should be noted that Patients with bronchial asthma of mild and severe severity had different way and did not have the same degree of deficiency of the immune system.

**Conclusion:**

These data suggested that apoptotic factor of lymphocytes may play an important role in controlling immunity of patients with atopic bronchial asthma.

## Introduction

Each pathological process resembles a stereotypical reaction of the organism due to the action of various pathogens. Although there is a genetic and immunological specificity, all species highly organized (including humans) show practically the same stereotyped responses. Programmed cell death (PCD), which is similar to a natural physiological process [1-3] is a very illustrative example of the stereotypical reactions. An important progress in the study of the PCD concern the morpho-biochemical changes observed during apoptosis [4-6]. In this respect, with the current classification of programmed cell death we can note: PCD of type I- apoptosis, PCD of type II - autophagy and necrosis as PCD of type III [7]. If before, the decisive role of PCD was attributed to induce this process (physiological: during apoptosis, supra-physiological during necrosis), now the differences from apoptosis and necrosis of the surrounding cells and the organism are in the foreground. On the other hand, the process of autophagy in normal cells is a possibility of renewal of organelles [8-10]. It was established that autophagy plays a vital role during embryogenesis and in the post-embryonic metamorphosis [11]. The deregulation of this process plays an important role in many diseases such as: neurodegenerative diseases (Alzheimer’s and Parkinson’s) [7,10,12], myodystrophy and cardiomyopathy diseases [13,14], the aging and infections [12] and malignant tumors [7]. The intensification of the study on the process of cell death is due to the fact that there are several methods existing nowadays available to record the various manifestations of PCD and to analyze molecular mechanisms [15] which are tightly related to mechanisms of other important events (eg cell activation and associated biological signaling). The study of PCD proved to be productive and fruitful for the understanding of a certain number of important processes, including immune homeostasis and oncogenesis. In connection with the phenomenon of PCD, it was necessary to review a certain number of conceptual data of pathophysiology. In eukaryotes, PCD was previously considered as a negative process in view of the importance of identifying the phenomenon of necrosis. Nowadays, we have a better understanding of PCD: on the one hand, the death of cells in the body is seen as a natural process, and the existence of a multicellular organism requires a balanced relationship between life and death. Nevertheless, the role of apoptosis in the development of the pathological process is less obvious. It seems that this form of cell death (as opposed to necrosis) is not an indispensable component of the typical pathological process, but rather the malfunction of apoptosis is the cause of a certain number of diseases [6,16,17]. Thus, the relevance of this problem is defined by a correlation of the malfunction of PCD process with most diseases, including autoimmune diseases. The identification of the mechanisms of deregulation of the PCD associated with some specific diseases allows understanding the etiopathology of these diseases. The goal of our research was to determine the immunological characteristics and the biochemical and morphological parameters of PCD of lymphocytes of patients with atopic bronchial asthma (ABA) according to their degree of severity.

## Materials and methods

### Patients and blood sampling

The study was carried on the peripheral blood from relatively healthy individuals (n = 21) and asthmatic patients (n = 92). The group of patients was composed of individuals with different severity of asthma: 38 patients of mild severity with an average of 39 +/- 5 years, 20 patients of moderate severity (42+/- 5) and 34 patients of severe severity (42 +/- 5). At the time of blood collection, patients were hospitalized in the detachment of Pneumology and were not treated with glucocorticoid. All the donors were non smokers and were selected after consent. The collection of venous blood from donors was done in the morning before taking breakfast. The diagnosis of atopic bronchial asthma was established by medical doctors on the basis of data from the allergic anamnesis, the results of allegen skin prick test, epidemiology, and the experiments of nasal provokers and inhalers. The work was conducted under the rules of the Committee of Ethics in the Laboratory of Clinical Immunology and Allergy of RKB.

### Detection of antibody - antiDNA by enzyme immunoassay

The determination of the rate of antibody anti-double stranded DNA was performed by enzyme-linked immunosorbent assay (ELISA) [18], previously optimized in the laboratory of the State University of Kazan. The lyophilized DNA of erythrocyte of chicken was used as antigen. The blood serum samples were incubated for 40 minutes at 56 degrees of Celsius to inactivate the proteins of the complement system and to dissociate the immune complex. For the detection of the antibodies associated with DNA in the wells of the ELISA plate, peroxidase-conjugated to antibody against human anti-IgG was used. The response of the coloring of ELISA reaction was detected by spectrophotometer “Multiskan” in units of optical density at a wavelength of 492 nm.

### Determination of extracellular DNA concentration

The determination of the concentration of extracellular DNA was performed by the fluorimetric method with the aid of the colouring Hoechst 33342 (bisbenzimide) as fluorochrome. The level of fluorescence in solutions was measured in quartz cuvettes with 0.6 ml of volume using a fluorescence spectrophotometer “Hitachi MPF-4” (Japan) at an excitation wavelength of 358 nm and an emission wavelength of 458 nm. To determine the rate of fluorescence of DNA in solution, we took into account the fluorescence of Hoechst solution (0.24 μg) contained in a TNE buffer.

### Determination of circulating immune complexes (CIC) by the method of precipitation with polyethylene glycol (PEG)

The rate of CIC in the serum was determined by the method based on the selective precipitation of antigen-antibody complexes in 4.17% PEG having a molecular weight of 6000, followed by a photometric determination of the density of the precipitate.

### Separation of CIC by the precipitation method with PEG

to obtain CIC from the blood serum of patients with atopic bronchial asthma, 0.5 ml of serum was incubated with 0.5 ml of 7.5% PEG in 0.1 M borate buffer for 1 hour at 4°C. The precipitate was washed twice with 3.5% PEG in 0.1 M buffer, and centrifuged at 2500 rpm/min for 20 min at 4°C. And then the sediment suspended in 0.5 ml of phosphate buffered saline (PBS) was added.

### Isolation of T-lymphocytes

Lymphocytes were isolated by the standard method of zonal centrifugation proposed by Patel et al. [19] with a mixture of Ficoll-verograffin (ρ = 1077 g cm-1) [20,21]. This method consists of isolating 95% of T- lymphocytes and their viability was determined by the trypan blue exclusion method [22].

### Culture of lymphocytes

The cells obtained (2 × 10^6^) were diluted in 1 ml of the RPMI-1640 medium in plank made of plastic with flat bottom (Nung), then supplemented with 10% serum of foetal calf and 10 μl of L-glutamine (200 μg/ml) (Flow) [23]. The cells were cultured with or without dexamethasone (final concentration of 10^-4^М) and then samples were incubated in СО_2_-incubator (5% СО_2_) for 1 - 6 days [24,25].

### Ultrastructural study

To study the morphology of the cells, cells obtained after zonal centrifugation were precipitated successively in 2.5% glutaraldehyde and 1% OsO4 for 1 hour [26]. The sample was then immersed in Epon 810 after being dehydrated with ethanol (30-96 degrees of Celsius), in acetone and propylene oxide. Then the cutting was realized with the aid of ultra-microtome LKB-3 followed by the observation with an electronic microscope (Hitachi 125, Japan) [27] after placing the material in the uranyl acetate and lead citrate for the contraction.

### Determination of apoptosis

the quantification of apoptosis of lymphocytes was performed by the method of flow cytometry on FACSCalibur device (“Весton Dickinson”) with the use of some parameters, such as the determination of the fragmentation of DNA by the iodide propidium (IP) (“Sigma”) [28], the translocation of membrane phosphatidylserines to the surface of lymphocytes using merocyanine (МС540), the measuring of the variation in mitochondrial membrane potential of lymphocytes according to the fluorescence intensity CMX-Ros (“Molecular Probes”) [29]. More than 10, 000 cells were counted on each variant of the experiment.

### Statistical analysis

the analysis was performed using Excel program and Statistics 5.0. Comparison of ranges of variation was done with non-parametric criteria of Kryckaya Yollica and T- criteria of Manna Yitni. The authenticity of the frequency difference of the indices encountered was determined using the method of Fischer and *t*-test and partly with correction of Bonfferoni. The correctional analysis was performed by the method of Rank and Cpirmena-rs.

## Results

### Immunological aspects

#### *The content of antibodies against double-stranded DNA and the content of CIC in the blood of patients with ABA*

**Figure 1:**
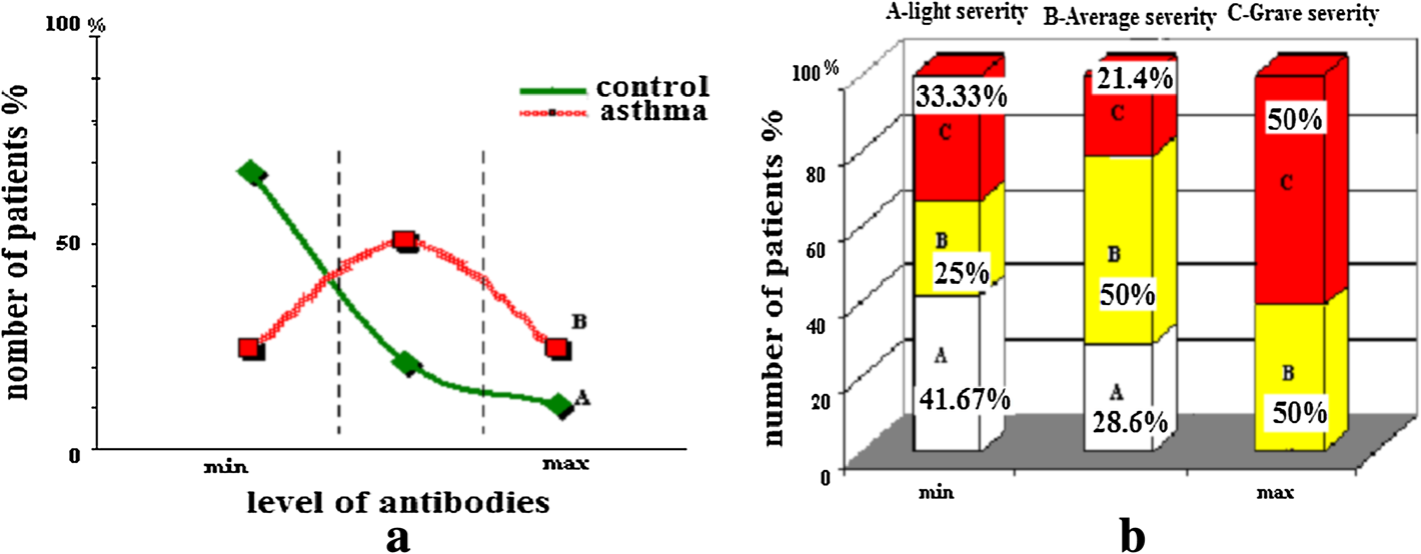
The distribution of antibodies – anti DNA complex in relatively healthy donors and asthmatic patients: - (a) the distribution of the level of antibody - anti DNA in the serum of donors, (b) - Distribution of the rate of antibody according to the degree of severity of asthma (mild, moderate and severe) in percentage.

**Figure 2:**
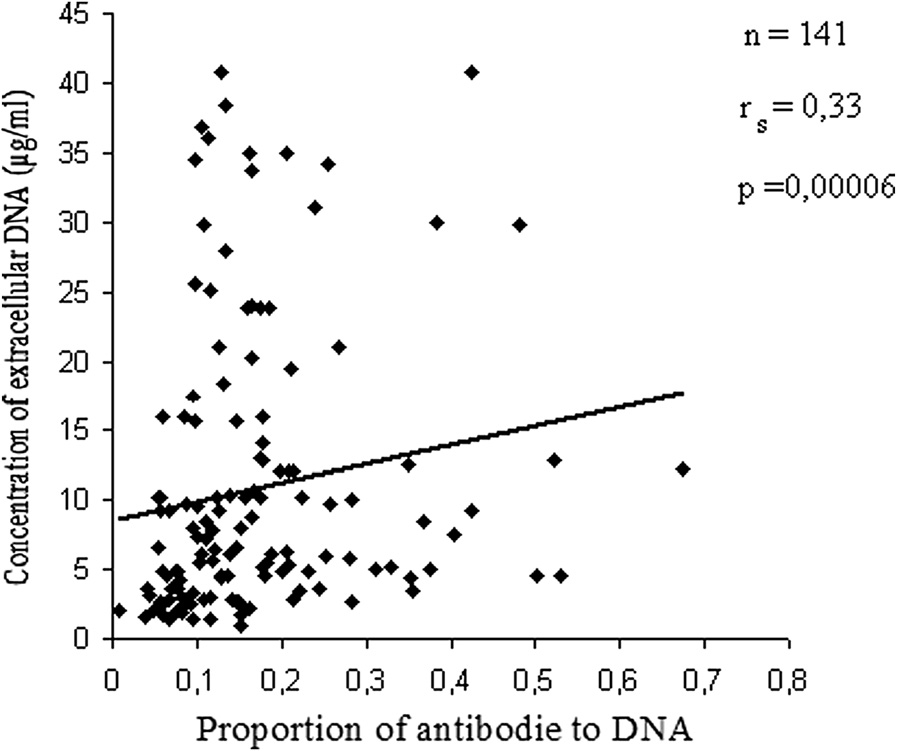
Relationship of dependence between the concentration of extracellular DNA and auto-antibodies anti DNA.

**Figure 3:**
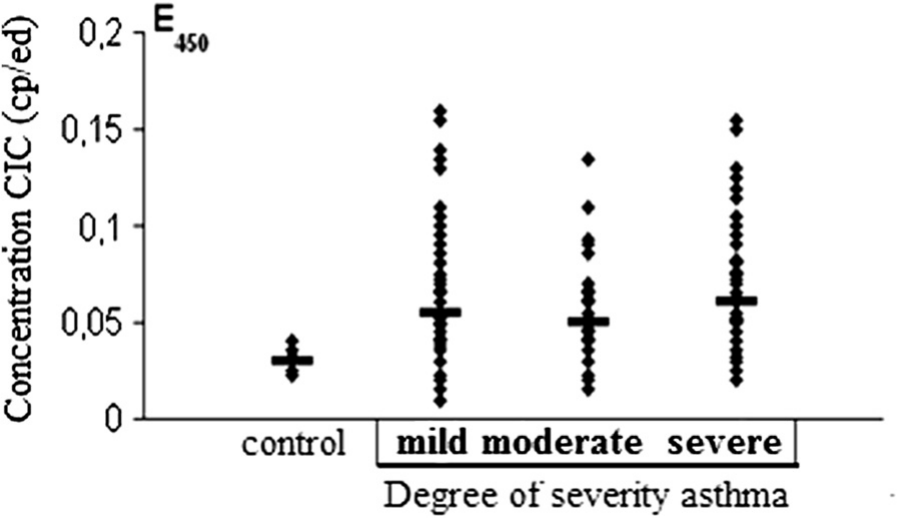
**Concentration of the circulating immune complex in the serum of healthy relatively donors and asthmatic patients according to the degree of severity (mild, moderate, and severe).** Median (Me) 0.03 for relatively healthy donors and 0.06 for asthmatic patients.

**Figure 4:**
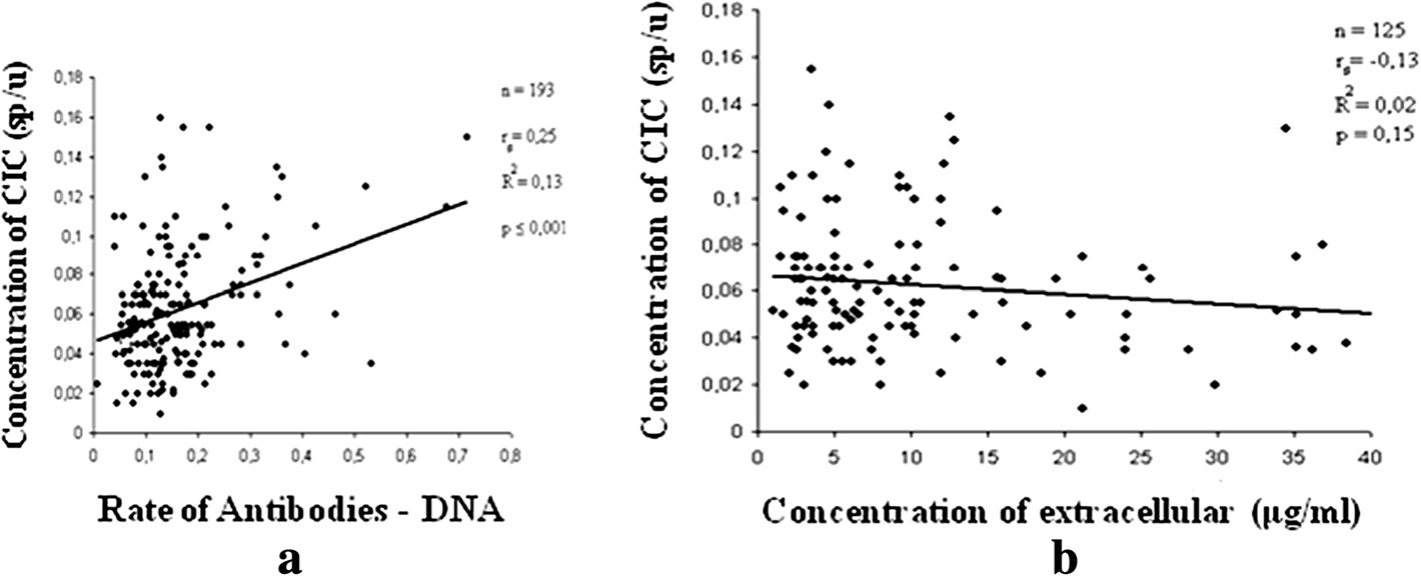
Determination of dependency relationships between immunological markers of patients with asthma: a- The dependency relationship between the concentration of CIC and the rate of antibodies anti-DNA, b- Dependency relationship between the concentration of CIC and the concentration of extracellular DNA in the blood serum of patients with atopic bronchial asthma.

The rate of antibodies against double-stranded DNA in the serum of patients with ABA and relatively healthy donors was examined using enzyme immunoassay (Figure 1). A minimum level of antibodies in all patient groups was observed (Figure 1b). With the method of correlational analysis we established a direct relationship of dependence between the high level of IgG antibodies – anti DNA and the severity of asthma (p = 0.0005). A significant and direct relationship of dependence between the level of extracellular DNA levels and that of antibodies (IgG аnti-nDNA) was also established (Figure 2). The attention was on the value of p (0.00006), reflecting the strong correlation between its significant data. The results of Figure 3 show a significant increase (p < 0.05) of the concentration of circulating immune complexes (CIC) in the blood of asthmatic patients compared with controls. And the concentration was significant in asthmatic patients with serious severity. In determining the size of the CIC, we observed that 85.6% of patients with bronchial asthma had CIC of small and medium size in the blood. On the other hand, in relatively healthy individuals, CIC of large size were recorded in 86% of donors and while 14% had CIC of medium size. Thus, the results of this study showed an increase in the concentration of CIC in asthmatic patients with a change in the size of the CIC towards the dominant formation of CIC of small and medium size and this rate of distribution was related to the severity of asthma. Using the method of correlational analysis, we have established with certainty a direct dependency relationship between the formation of CIC and antibodies to native DNA (Figure 4a) and on the other hand, the concentration of CIC was inversely proportional to the concentration extracellular DNA.

#### *Programmed cell death of lymphocytes of patients with ABA*

**Figure 5:**
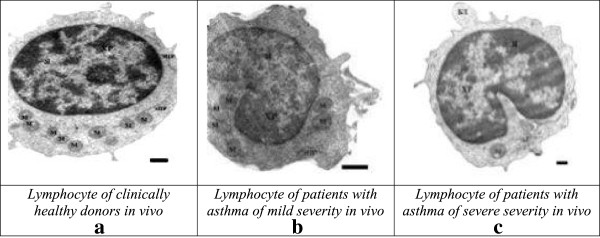
Comparative study of Ultrastructure of lymphocytes of clinically healthy donors (a) and of patients with asthma according to the degree of severity: (b)-mild severity (c) severe severity in vivo.

**Figure 6:**
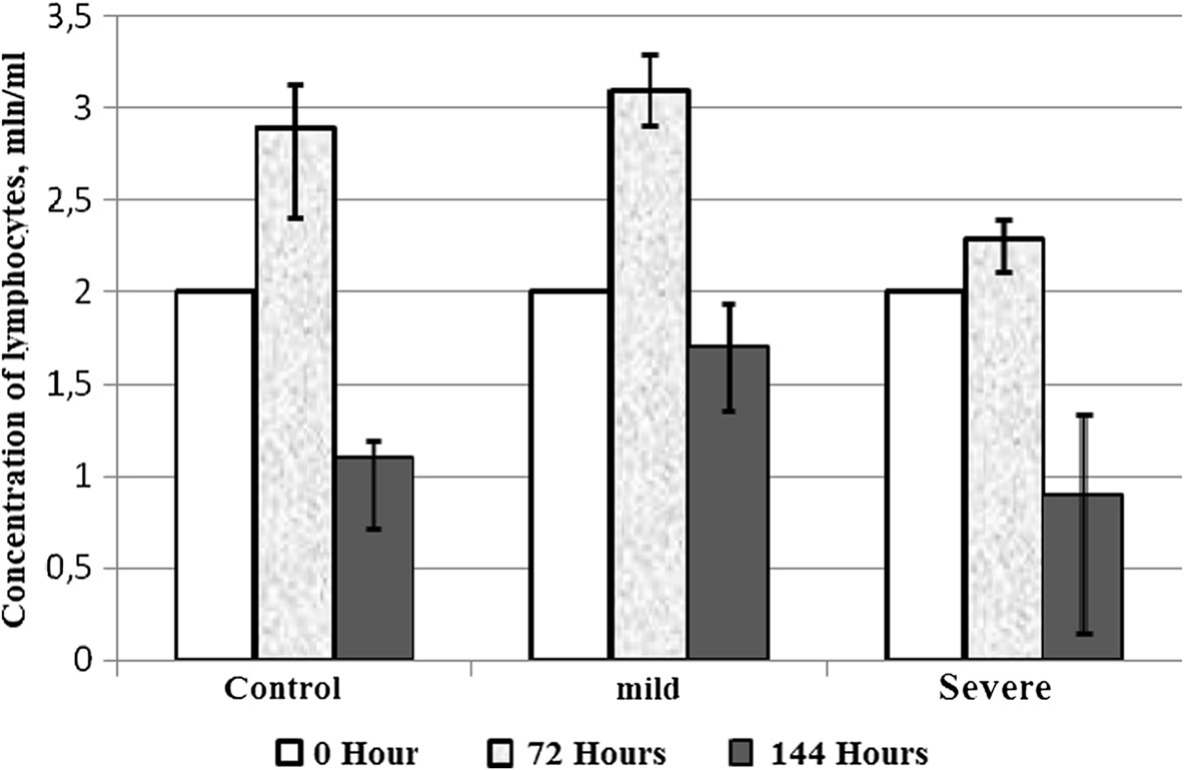
Evolution of the concentration of lymphocytes of clinically healthy donors and asthmatics with mild and severe severity after 0, 3 and 6 days cultures in vitro.

**Figure 7:**
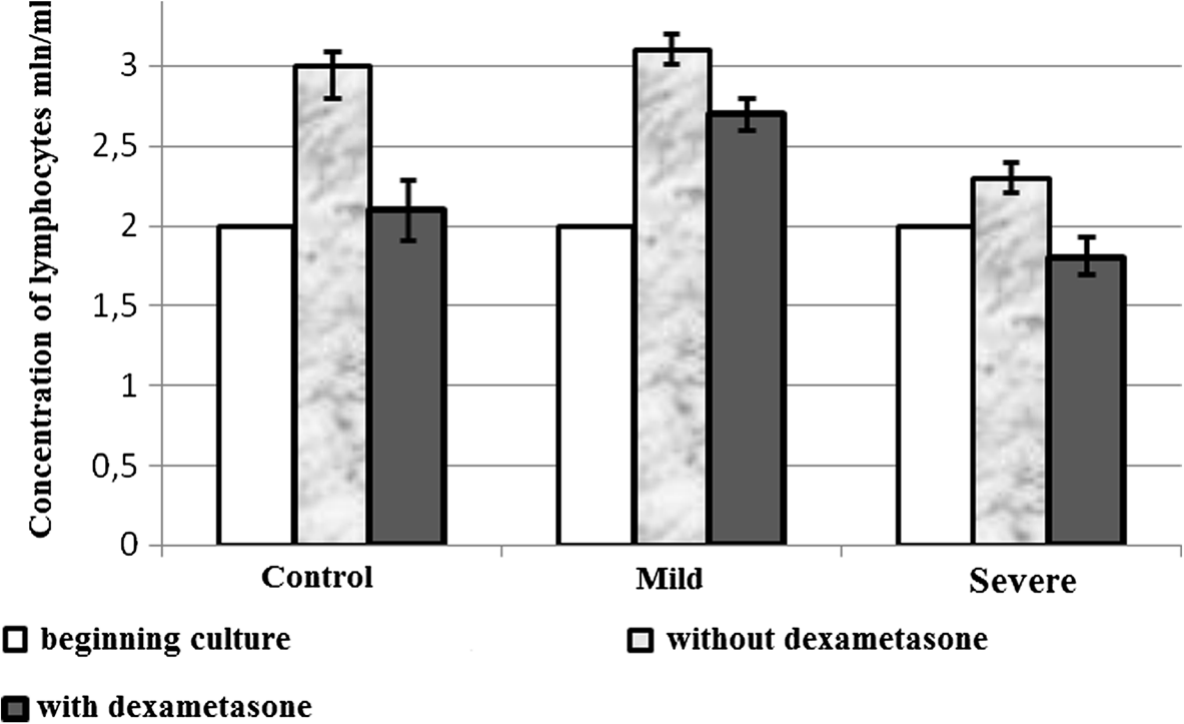
Variation of the number of lymphocytes (mln/ml) of clinically healthy donors (control) and of patients with asthma (mild and severe severity) during 3 and 6 days of culture with or without dexamethasone in vitro.

**Figure 8:**
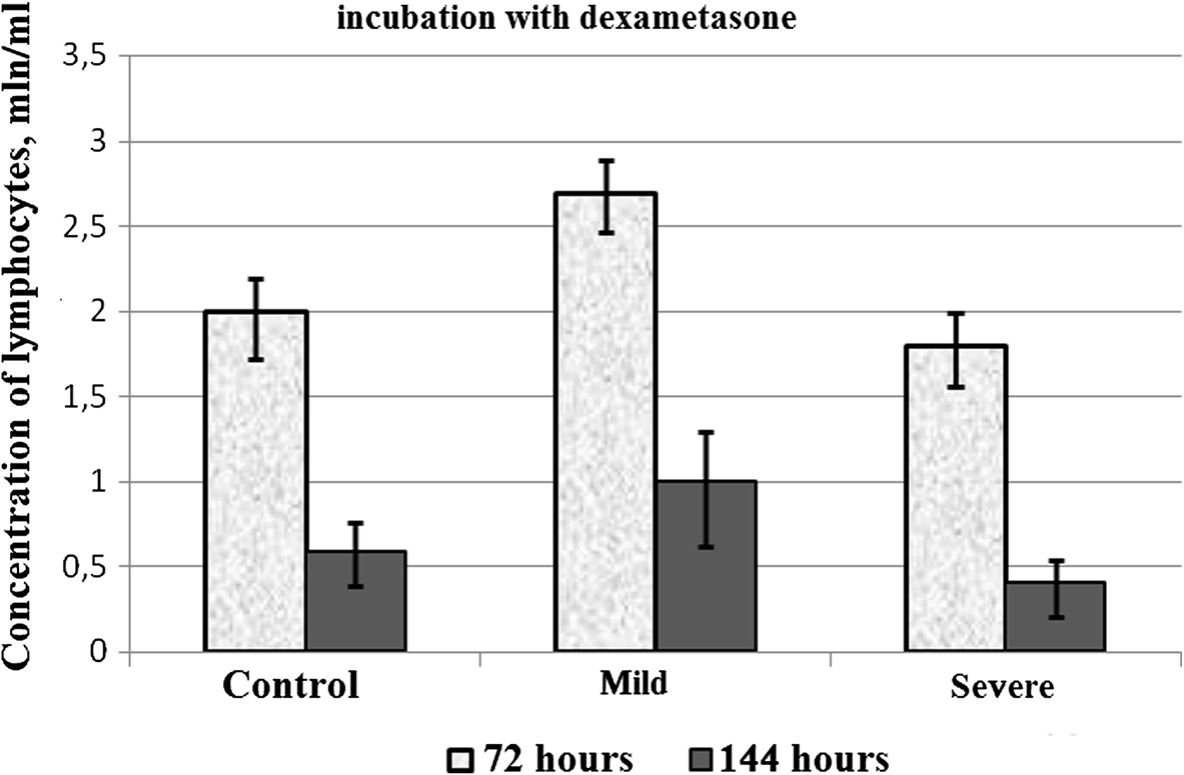
Comparative study of evolution of lymphocytes of clinically healthy donors and patients with asthma of mild and severe severity after 3 and 6 days of culture in the presence of dexamethasone.

**Figure 9:**
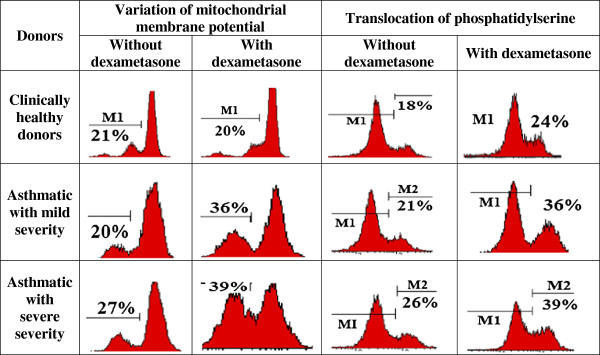
Cytofluorogramme showing the quantification of the apoptosis of lymphocytes of relatively healthy donors and of asthmatic with mild and severe severity by using some parameters such as the translocation of phosphatidylserine at the surface of lymphocytes with MC540 and the variation of mitochondrial membrane potential according to the fluorescence intensity of CMX -Ros during 3 days of culture with dexamethasone.

**Figure 10:**
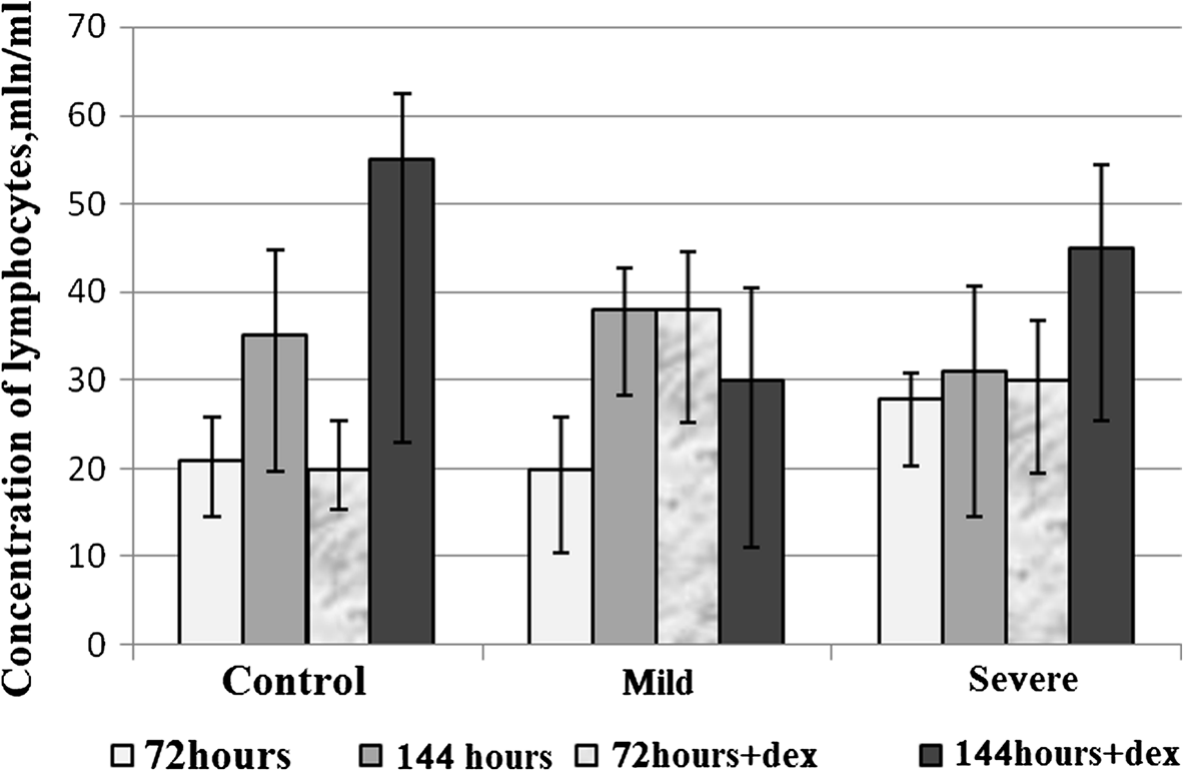
Variation of mitochondrial membrane potential of lymphocytes of relatively healthy donors and patients with atopic bronchial asthma (mild and severe severity) after 3 and 6 days of culture with or without dexamethasone in vitro.

**Figure 11:**
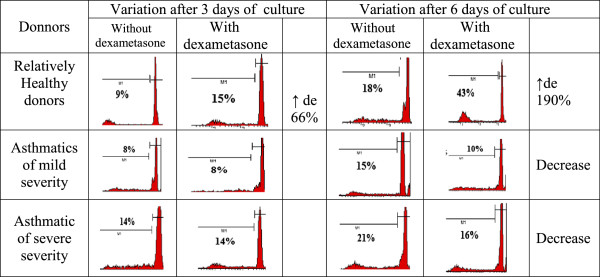
Cytofluorogramme showing the quantification of the apoptosis of lymphocytes of relatively healthy donors and of asthmatic with mild and severe forms by using some parameter such as the determination of DNA fragmentation with propidium iodide after 3 days and 6 days of culture without or with dexametasone.

**Figure 12:**
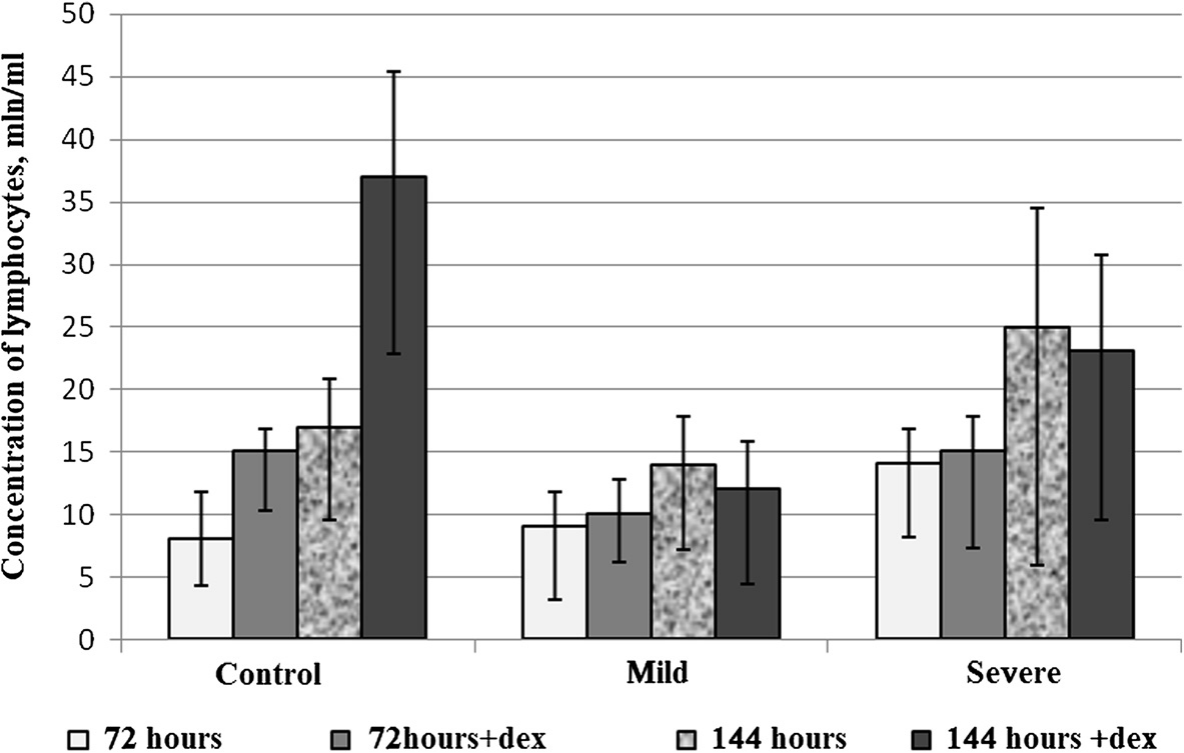
Variation of the concentration of lymphocytes during the advance phase of apoptosis in clinically healthy donors and asthmatics with mild and severe severity after 3 and 6 days of culture with or without dexamethasone.

Given that changes were observed rapidly at cellular level, we found interesting the study of biochemical and morphological parameters of lymphocytes of patients with asthma in vitro after reviewing the immunological aspects. Therefore, we conducted a comparative analysis of morphological characteristics of lymphocytes of relatively healthy donors and patients with asthma of mild and serious severity. Thus, with the aid of transmission electron microscopy morphological differences were identified between the lymphocytes of control group and the lymphocytes of patients with asthma. The electron microscope revealed morphological and structural changes in lymphocytes especially in the nucleus, the chromatin and plasma membrane of patients with asthma and this according to the severity degree of the disease. Cells with a modified structure were observed: on the cell surface outgrowths appeared on the cell membrane, deep invaginations and chromatin condensation and orientation of the nucleus towards the periphery with intussusceptions. The structure of the lymphocytes of the peripheral blood from asthmatic patients with serious severity differs from the structure of the cells of clinically healthy donors and that of asthmatics with mild severity (Figure 5). The buddings known as the blebbing were observed on the cell membrane [30]. Besides, some cells contain phagosomes with cellular debris inside which are generally characteristics of autophagic cells (Figure 5a,b). The study of apoptosis in vitro now provides a better understanding of malfunction of the molecular processes during asthma. In the culture media most of the cells die by apoptosis. But our present study showed that this statement is not always true in asthmatics. To study the biochemical parameters in vitro, we compared the level of lymphocytes for 3 and 6 days of growth in the presence or in the absence of dexamethasone which is an inductor of apoptosis. As shown in Figure 6, the rate of lymphocytes increased after 3 days of growth at all the groups studied, particularly in clinically healthy donors and asthmatic patients with mild severity. On the other hand, after 6 days of growth there was a decrease in the rate of lymphocytes at all groups studied compared to those observed after 3 days of culture (Figure 6). The use of dexamethasone as inductor of apoptosis resulted in an increase of 5% (Figure 7) of the level of lymphocytes in clinically healthy donors after 3 days of culture and a decrease of 70% (compared to 3rd day) after 6 days of culture (Figure 8). The structure of these lymphocytes underwent significant changes, which may indicate an inhibition of mitotic activity of the cells of clinically healthy donors in the presence of dexamethasone. The lymphocytes of asthmatic patients with mild severity in the presence of dexamethasone continue to proliferate with a slight decrease in the proliferative activity of its lymphocytes, which could explain the death of its cells following apoptotic induction. After 6 days of culture, there was a decrease in lymphocytes of patients with mild severity (Figure 8). More attention should be paid to the lymphocytes of asthmatic patients with serious severity: if in the absence of dexamethasone, the level of lymphocytes increased by 15% while in the presence of the apoptotic inductor this level declining by 10% after 3 days of culture as compared to the level before the cell culture. With cytometry method the quantification of apoptosis of lymphocytes with the use of some biochemical parameters was performed. During the study of the mechanism of PCD, great importance was given to mitochondria as it releases a large amount of biologically active substance necessary for the transition from apoptosis to the final and irreversible phase [30]. From the analysis of Figure 9, a correlation between the number of cells with a decrease of mitochondrial membrane potential and the expression of phosphatidilserine on the membrane wall of lymphocyte of clinically healthy donors and asthmatic was observed. After 3 days of culture, no significant difference was found between the numbers of lymphocytes with a decrease in membrane potential which underwent a translocation of phosphatidylcholine on the cell membrane (Figure 9). The level of lymphocytes with a decrease in membrane potential in clinically healthy donors has increased by 70% and 110% in asthmatics with mild severity. On the other hand, this level is not as expressive in asthmatic of serious severity after 6 days of culture. The incubation of lymphocytes in the presence of dexamethasone leads to a decrease in the size of mitochondrial membrane potential in some lymphocytes but the dynamics vary considerably from one group to another. In the presence of dexamethasone, a slight resistance of lymphocytes of clinically healthy donors compared to a spontaneous apoptosis was observed (Figure 10). After 3 days of incubation with dexamethasone, that the number of lymphocytes with decreased mitochondrial membrane potential was higher in asthmatics compared to clinically healthy donors was recorded. The lymphocytes of patients with ABA showed resistance to the development of apoptosis while clinically healthy donor lymphocytes were characterized by a progressive increase in PCD after 6 days of culture. A small demonstration of early signs of spontaneous apoptosis and induced lymphocytes of patients with asthma of mild and serious severity after 6 days of culture in vitro might lead us to think of a disruption of this process of PCD of type 1. The use of iodide propidium revealed cells with fragmented DNA (Figure 11). According to some authors, the morphological characteristics of DNA fragmentation were characterized by an invagination of the nuclear membrane and condensation of chromatin at the nuclear periphery level. In vitro studies showed no significant difference in DNA fragmentation of lymphocytes (Figure 12). Even after 6 days of incubation, the DNA fragmentation of lymphocytes of asthmatics was less pronounced compared to controls. The results showed that there were cells with fragmented DNA both in asthmatics and in clinically healthy donors. Culture of cells without dexamethasone (spontaneous apoptosis) for 3-6 days resulted in a fragmentation of the DNA of lymphocytes (Figure 12). The number of cells with fragmented DNA almost doubled in the culture medium of relatively healthy donors (9% -18%) and in asthmatics with mild severity this variation was 8% to 15% while it was 14% to 21% in asthmatics patients with serious severity. When comparing among themselves, the development of the process in some cultures (for 3 and 6 days) in the presence of dexamethasone, there was a 3-fold increase in the number of cells with characteristic traits of the final phase of apoptosis (ie 187%) in relatively healthy donors (Figure 12) and 25% (from 8% to 10%) in asthmatics with mild severity and 14% (ie from 14 to 16%) in asthmatics with serious severity. We followed the dynamics of the induction of spontaneous apoptosis and apoptosis induced by dexamethasone of lymphocytes in vitro in different groups studied and it was found that the lymphocytes of relatively normal donors responded to the induction of spontaneous apoptosis and induced by dexamethasone with a significant increase in the number of apoptotic cells in the final phase of 67% after 3 days of culture and 190% after 6 days of culture (Figure 12). As the results show, the asthmatic cells demonstrate a resistance to apoptosis induced by dexamethasone.

## Discussion

In a physiopathological context, asthma is a chronic inflammatory disease of the respiratory airways [31] and the choice of the study of programmed cell death is particularly due to the fact that these lymphocytes play an irrecusable role in the pathogenesis of asthma [17,32,33]. Despite the plentiful data on asthma, the concept of PCD in the pathogenesis of asthma is and remains misunderstood and controversial [34,35]. Increasing interest in this process of PCD [36] in recent years was fundamental to conduct this present study. During the development of asthma a multitude of Immunogenetic mechanism and cells of immune systems might be involved [37]. The results of immunological studies suggest a number of changes in the functional state of lymphocytes in patients with asthma. In 1995 Szczeklik et al. [38] while studying the autoimmune status of patients suffering from Bronchial asthma, found in the blood some antinuclear antibodies, in any case, none of the patients got some antibodies with _N_DNA, however, the quantity of autoimmunity of the patients suffering from bronchial pulmonary system reveal the advantage of the synthesis of specific organ antibody, for example _N_DNA and/or _DN_DNA autoantibodies [39]. Therefore, the level of IgG anti -_N_DNA increased significantly according to the degree of severity of asthma (Figure 2) and given that IgG have the ability to be locked up in the tissues, one could think of a lesion of target tissues in progressive course with the severity degree of the disease. It was revealed in patients with asthma the significant presence of CIC of average size and considered as the most pathogenic of CIC which was formed during a slight plethora of antigens. In relatively healthy donors, a process of enlargement of chronic CIC was observed and this in accordance with a proportional relationship of the level of antigen and antibody corresponding to their elimination. It should be noted that we recorded a direct relationship of dependence between the concentration of CIC and the level of antibodies against DNA and inverse dependence between the concentration of CIC and the concentration of extracellular DNA in the blood (Figure 6). This leads us to suggest the absence of a relation of equivalence between the antibody and the antigen during the progression of the severity degree of asthma. The existence of CIC in the blood is a normal and natural phenomenon of immune reactions. But their high level constitutes a means of diagnosing the pathology. Circulating immune complexes of large size are less soluble and can be easily removed by macrophages unlike CIC of medium and small size. They dissolve easily, making their removal difficult, which explains the CIC concentrations of medium and small size in asthmatics with serious severity. Therefore, the conception of the role played by the immune process in the pathogenesis of asthma is widely acknowledged, however, the importance of indicators of diagnosis and individual prognosis of the immune system remains poorly studied and in particular the accumulation in biological fluids IG antibodies - anti DNA, abzymes with DNAse activities, extracellular DNA, and CIC according to the degree of severity of the disease. The results obtained in this study allow us to suggest that these markers play a vital role in the pathogenesis of autoimmune process during asthma. And it is not excluded that these immunological characteristics stemmed from other natural physiological process. Thus, the observed difference underlined in the mitotic activity and the variation of the level of lymphocytes in culture of clinically healthy donors and asthmatic shows varying degrees of cell survival according to the severity of the disease. From the results of our work we could note that, based on a slowing of cell growth of lymphocytes from asthmatics with severe severity, the lymphocytes of asthmatics with mild severity are characteristic of lymphoproliferous activity in vitro. For the researches on asthma we do not often take into account the severity of asthma. Patients with asthma of mild and severe severity have different backgrounds and do not have the same degree of deficiency of the immune system. Failure to take into account the degree of severity could lead to false reasoning of statistical analysis. We found after our study that PCD of lymphocytes of patients manifested differently based on their degree of severity. Further to the adoption of the concept of PCD of type 1, the death of lymphocytes under the influence of certain doses of glucocorticoids was considered a classic model of apoptosis [40,41]. Recently data were published on the sensitivity of subpopulations of lymphocytes of peripheral blood particularly cytotoxic and NK lymphocytes at a high dose of dexamethasone [42,43]. The use of systemic glucocorticoids increases systematically the number of lymphocytes in the dipodiploide area. This confirms the hypothesis that the use of corticosteroids inhibit cytokine production (Ile 3, 4, 5) which keeps the high level of Bcl-2 anti apoptotic protein) and consequently leads to an acceleration of apoptosis lymphocytes [24,44]. On the other hand, studies showed that the use of dexamethasone leads to a decrease in the number of migrating cells in the lungs after inhalation of specific allergens [45]. The dexamethasone as inductor of apoptosis is expected to influence the number of proliferating cells especially their reduction [46,47]. But according to our data, the lymphocytes of asthmatic patients with mild severity showed resistance to glucocorticoids followed by active proliferation of its cells. There was also a decrease in the number of cells in the late phase of apoptosis. This may be due to a malfunction of the expression of DNase responsible for DNA degradation. It is conceivable in view of these results that there are indeed particularities in the course of apoptosis, in asthmatics and this according to the degree of severity of the disease. However, even if the conditions of the body seem to be met to artificially induce apoptosis, the results obtained could not totally be those of internal conditions. Several parameters can influence the results. Researches on apoptosis still continue nowadays, it may be other unknown physiological parameters which determine the course of this process, of which the absence would influence the results. Asthma can also be linked to a genetic predisposition, and even from a race to another, differences can be observed. Thus, results on the morphology of lymphocytes of asthmatics were obtained. And most of lymphocytes had specific morphological characteristics. Sometimes, cells with characteristics of autophagosomes autophagy were found [29,48]. The presence of autophagy could explain the decrease of apoptotic cells in asthmatics with mild severity. And the PCD is universally prevalent in the world of multicellular organisms, and affects all types of tissues. It operates according to the biochemical and morphological parameters strictly defined and do not depend on the causes leading to the initiation of this process. In addition, the study of PCD is very productive to understand a certain number of important processes including immune homeostasis. Finally, according to new data, it has become essential and indispensable to review a certain number of conceptual bases of physiopathology and immunopathology.

## Conclusion

Autoimmunity and Programmed Cell Death are a highly conserved and integrated response in normal physiological processes playing a vital role in the pathogenesis of various diseases, especially in the development of asthma.

## Competing interests

We have no competing interests.

## Authors’ contributions

ZIA, CAV, LBM, SAA designed the study. CAV, YVS, VE, CBC participated in the technical work and the acquisition and interpretation of data. CBC, AN, SOK, SOK, CAV evaluated the literature. CAV, LBM, ZIA, YVS, YVS carried out the experiments of this study. SOK, LBM, VE, SAA, SOK, CAV and ZIA have given final approval of the version to be published. All authors have read and approved the final manuscript.
